# Body Composition Assessment Provides Prognostic Information in Patients With Cancer Affected by Chronic Graft vs. Host Disease

**DOI:** 10.1002/jcsm.13759

**Published:** 2025-03-28

**Authors:** Asmita Mishra, Ram Thapa, Kevin Bigam, Martine Extermann, Rawan Faramand, Farhad Khimani, Xuefeng Wang, Vickie Baracos, Joseph A. Pidala

**Affiliations:** ^1^ H. Lee Moffitt Cancer Center Tampa Florida USA; ^2^ University of Alberta Canada Edmonton Alberta Canada

**Keywords:** abdominal visceral fat, graft vs. host disease, sarcopenia

## Abstract

**Background:**

Additional tools are needed to assess mortality risk among patients with cancer. Patients with chronic graft vs. host disease (cGVHD) after allogeneic haematopoietic cell transplantation (HCT) represent a high‐risk cancer population with mortality risk explained by cGVHD severity, but also informed by baseline comorbidities, functional status before and after HCT, and cumulative toxicity from the procedure and its complications. Radiographic body composition metrics from CT scans have previously shown association with complications in other populations.

**Methods:**

We examined a single‐centre consecutive series (2005–2016) of HCT recipients with cGVHD and CT‐scans immediately proximal to cGVHD diagnosis to investigate association of radiographic body composition measures and mortality. Skeletal muscle index (SMI) and fat index (FI) were quantified on CT imaging at the 3rd lumbar (L3) and 4th thoracic (T4) vertebra. SM Hounsfield units (HU) were obtained to evaluate SM density. Cut points for SMI were from literature and cut points for FI were established by sex‐specific optimal stratification.

**Results:**

A total of *n* = 113 patients met the inclusion criteria for this analysis, aged 51.2 ± 10.5(SD) years and predominantly male (*n* = 71, 63%) and diagnosed with NHL (*n* = 110, 97%). Onset cGVHD NIH overall severity was mild in *N* = 56 (49%), moderate in 44 (38%) and severe in 15 (13%), with median time to cGVHD onset after HCT of 173 days [IQR 122;295]. A CT scan at 77 days [IQR 33;202] post HCT was selected for analysis. In multivariate analysis, CT‐defined body fat ≥ 35% was independently associated with increased mortality (HR 2.094 (95% CI 1.060, 4.136), *p* = 0.033) overall. Patients of male sex had higher FI than females and showed a more prominent association between high FI and mortality. SMI as well as other indices of adiposity were not associated with survival in multivariable analysis including BMI, sarcopenic obesity and low skeletal muscle radiodensity. In exploratory analyses, we demonstrated similar results per CT chest at T4, suggesting possible future application to a larger HCT population.

**Conclusions:**

These data support that radiographic body composition measures provide prognostic information among patients with cancer affected by cGVHD post‐HCT and suggest that high body fat % is a promising candidate for future study. These findings suggest that low skeletal muscle mass alone does not predict for poor outcomes in HCT patients with cGVHD as previously described in other cancers. Independent validation of this work is needed, including further studies based on CT chest to enhance application to a larger HCT population.

**Abbreviations**
CT: computed tomography scan
FI: fat index
GVHD: graft vs. host disease
HCT: allogeneic haematopoietic cell transplantation
HU: Houndsfield units
L3: third lumbar vertebra
SAT: subcutaneous adipose tissue
SMA: skeletal muscle area
SMI: skeletal muscle index
SMR: skeletal muscle radiodensity
T4: fourth thoracic vertabra
VAT: visceral adipose tissue

## 1 Introduction

A growing body of evidence supports that body composition among patients with cancer holds important prognostic information that may both identify risk of mortality and suggest opportunities for interventions among individual patients. These measures of body fat and muscle proportions offer an integration of patient fitness and functionality, cancer associated cachexia, deconditioning and toxicity of cancer therapy and allied complications. Sarcopenia, or loss of skeletal muscle mass results in impairments in strength, functional limitations, physical disability, and potentially institutionalization [1, 2, 3]. While age‐associated decrease in physical activity is noted to play a predominant role in developing sarcopenia [4], several alternative mechanisms such as malignancy and associated treatment are also known to be involved in these changes [5]. Published data demonstrate reduced muscle strength, mobility, and endurance in young cancer survivors aged 20–30s that are comparable to healthy individuals aged 60–70 [6, 7]. Furthermore, presence of sarcopenia (detected by secondary analyses of oncologic images) increases morbidity and mortality in patients with malignant diseases [8, 9, 10, 11, 12, 13, 14], and has been reported in patients after transplant [15, 16]. Similarly, specific measures of total, intramuscular, visceral and subcutaneous adipose tissues are enabled by a radiological approach, and this has led to understanding of adiposity that extends well beyond the crude measure of BMI. However, while there is some suggestion that obesity is associated with poorer clinical outcomes, these relationships are not fully defined [17].

Allogeneic haematopoietic cell transplantation (HCT) is a widely used and potentially curative therapy for a broad range of hematologic malignancies and disorders, many of which preferentially affect older adults with other medical comorbidities or impaired functional status. Importantly, this curative potential is challenged by a loss of physical functionality [18, 19, 20, 21, 22], in part driven by a commonly occurring post‐HCT complication, chronic graft vs. host disease (cGVHD). This syndrome directly challenges already frail patients with cancer through cGVHD itself and toxicity of cGVHD therapy and is the leading late post‐HCT source of mortality, morbidity, disability, impaired quality of life, and prolonged duration of immune suppressive therapy [23, 24, 25]. As well, recent evidence supports a high prevalence of metabolic syndrome in patients with cGVHD [26]. Consensus criteria‐based cGVHD severity scoring is associated with risk of mortality, yet there are limitations in risk assessment: A large proportion of affected patients arrive at a moderate severity (rather than mild or severe) [23, 27], and through a diverse set of affected individual organ sites and severity that may have important distinctions regarding longer‐term prognosis. Thus, study of radiographic body composition in this population is of great interest, both to assess risk in this frail and high‐risk cancer population and to potentially inform current clinical risk stratification tools.

We hypothesized that altered fat and muscle body composition measures would be detected among cGVHD‐affected patients with cancer, and that these may provide prognostic information regarding mortality risk that ultimately could be used for risk stratification, education, and targeted interventions. To address this goal, we performed a retrospective analysis of abdominal CT‐based body composition measures among cGVHD affected patients with cancer post‐HCT, appealing to available CT studies performed proximal to cGVHD onset. In exploratory studies, we performed similar work using CT chest studies.

## 2 Materials and Methods

Ethical approval was granted by Chesapeake Institutional Research Board (Protocol number Pro00018503).

### 2.1 Population and Methods

This retrospective analysis appealed to available CT scans and clinical data. Acknowledging that body composition may change over time post‐HCT, we aimed to identify the most proximal post‐HCT scans to cGVHD development to better capture the current state of the affected subjects. With an expected median onset time to cGVHD of 5–6 months post‐HCT, we used 3‐month post‐HCT scans routinely obtained for re‐staging purposes among patients with lymphoma to address the research question. Thus, the main eligibility criteria for this analysis were the following: 1) consecutive adult patients ≥ 18 years of age, 2) receipt of HCT at the H. Lee Moffitt Cancer Center January 2005 through July 2016, 3) diagnosis of cGVHD post‐HCT and 4) have CT imaging done after HCT but prior to the onset of cGVHD. Outside of these core eligibility criteria, we did not impose any other direct exclusion criteria. For example, no other restrictions were placed on the included type of lymphoma or the HCT‐specific factors (e.g. conditioning regimen intensity, GVHD prophylaxis). For secondary analyses, we also included CT chest studies when available. Patient demographics obtained from medical records included metrics needed for 1) body composition analysis including age, sex, height, weight, and body mass index (BMI), and 2) transplant related demographic data including diagnosis (HL vs. NHL), performance status, previous chemotherapy regimens, conditioning chemotherapy intensity, match grade, HCT source, length of stay, and comorbidities.

### 2.2 Chronic GVHD Grading

Chronic GVHD organ involvement, severity and sub‐type were all assessed per established NIH Consensus Criteria [27]. For the purpose of this analysis, we focused on cGVHD features and severity at onset to benchmark this timepoint together with the CT scans closely preceding cGVHD onset. Additional established prognostic factors at cGVHD onset (platelet count, bilirubin, Karnofsky performance status, cGVHD onset type from prior acute GVHD) were also collected.

### 2.3 Body Composition Assessment by CT Imaging

CTs allows for precise and specific examination of muscle and adipose tissue (e.g. volume, density, and cross‐sectional area) [5, 28, 29]. For thoracic series, we identified a single axial CT image landmarked at the 4^th^ thoracic vertebra (T4); for lumbar series, a secondary single axial image at the 3^rd^ lumbar vertebra was selected. Vertebral bodies were selected at the level of the pedicles. All images were of 3 mm slice thickness, with a peak kilovoltage of 120 and current varying by patient within the standard algorithms within the SIEMENS CT scanners at this site.

**FIGURE 1:**
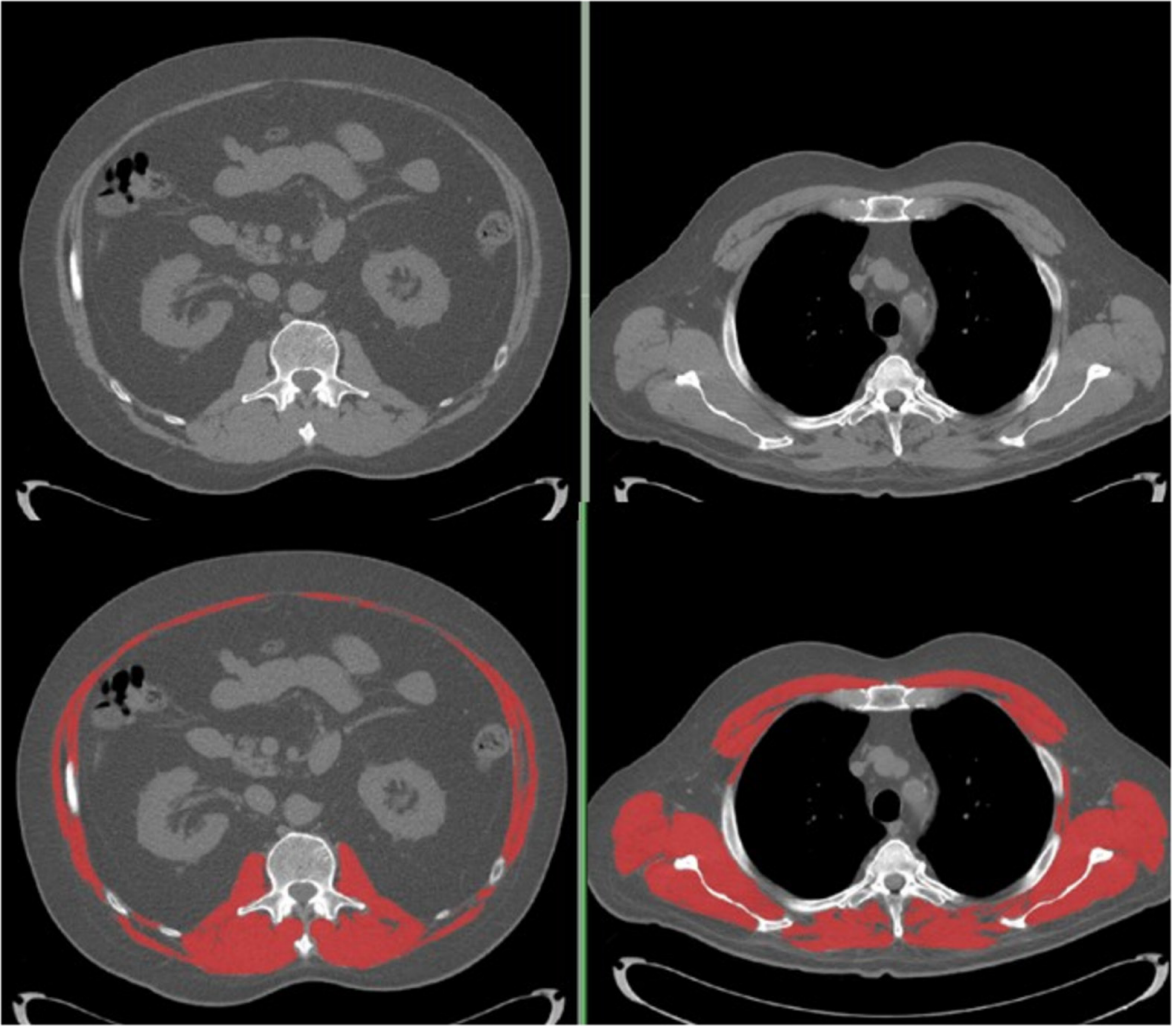
Representative cross‐sectional images of T4 and L3 CT scan slices: Left example lumbar images, Right example thoracic image. Lower panels: Images from upper panels with skeletal muscle annotated in red.

We quantified tissue cross‐sectional areas with SliceOmatic® (TomoVision, Magog, Quebec, Canada). Predetermined Hounsfield unit (HU) thresholds for muscle were −29 HU to +150 HU, −50 to −150 HU for visceral adipose (VAT) and −30 to −190 HU for subcutaneous adipose (SAT) [29]. Skeletal muscle area (SMA), mean skeletal muscle radiodensity (SMR) and areas of VAT and SAT were reported. Adipose tissue outside of the abdominal wall but inside the muscle fascia was included with SAT. Areas of VAT and SAT were summed to obtain the area for total adipose tissue at the L3 level (VAT is absent at T4). Example images are present in Figure 1 (T4, panel a; L3, panel b). Muscle cross sectional area was normalized for height^2^ and reported as skeletal muscle index (SMI, cm^2^/m^2^). Similarly, total adipose tissue area was normalized for height^2^ and reported as fat index (FI, cm^2^/m^2^). Body fat mass (kg) was determined as = (total L3 fat cross sectional area cm^2^ × 0.068) + 4.142 [29]; this value was divided by body weight to derive %body fat. Optimal stratification was utilized for determining cut points based on survival as previously described [12] for FI, as no such cut‐points have been previously reported in patients with cGVHD. To compare the incidence of sarcopenia with reports in the literature, sarcopenia severity (Class I moderate, Class 2 Severe) was defined by the lumbar SMI cutoffs as previously reported in solid tumour cancer cohort [30]. Sarcopenic obesity was defined BMI ≥ and SMI cutoffs defined previously [12].

### 2.4 Statistical Methods

Descriptive statistics were used to summarize and illustrate the studied body composition variables for this study, as well as other clinical variables. Candidate main body composition variables studied in this analysis included radiographic measures (SMI, FI, skeletal muscle Hounsfield units (HU)) as well as anthropometric measures (weight, body mass index (BMI)). Sex‐specific optimal cut‐points associated with mortality of different body composition variables were determined using the optimal stratification method [31], and these variables were then dichotomized by these optimal cut points to study association with survival outcome. Overall survival was selected as the primary endpoint of the study, given its unambiguous assessment of success or failure regarding HCT outcome. In univariate analysis, we studied the association between each body composition variable as well as chronic GVHD variables (age, platelet count, bilirubin, KPS, cGVHD overall severity score, overlap vs. classic cGVHD status, and HCT‐CI score at cGVHD onset) with overall survival assessed from time of cGVHD onset. We performed variable selection procedure with stepwise regression using AIC criterion to select multivariable model of overall survival. Chronic GVHD variables with *p*‐value < 0.2 from univariable analysis and all body composition variables dichotomized by optimal cut points were selected as candidate variables in stepwise regression for variable selection in the final multivariable model. The primary analysis proceeded as described using data arising from the L3 scan (*N* = 113 subjects), as this is the accepted standard. In secondary analysis, we performed the same analysis using CT chest T4 data.

## 3 Results

### 3.1 Study Population

A total of *n* = 113 patients were evaluable (Table 1). The median age was 51.2 ± 10.5 (SD) years, and patients were predominantly male (*n* = 71, 62%). Most patients underwent HCT for diagnosis of NHL (96%). Median onset time of cGVHD from HCT was 173 days [IQR 122;295]. Onset cGVHD NIH overall severity was mild in *N* = 56 (49%), moderate in 44 (38%) and severe in 15 (13%) with 84% of patients having history of prior acute GVHD. From pre‐HCT to time of cGVHD assessment there was a decrease (75% decreased to 57%) in patients with high performance status reflected by KPS score of 90 or greater. We also noted that prior to HCT 35% (*n* = 40) of patients had HCT‐CI score of ≥ 3 and we noted an increase in comorbidity burden at the time of onset of cGVHD with 55% (*n* = 63) having HCT‐CI score of ≥ 3, reflecting toxicity and increased morbidity after HCT procedure.

**TABLE 1:** Demographic and body composition summary of the study population.

Feature	All patients *N* = 113	Female *n* = 44	Male *n* = 69	*p*
Age (mean, SD)	51.2 ± 10.5	51.8 ± 11.5	50.9 ± 9.9	NS
Sex: Male (*n*, %)	71 (61.7%)			
HD vs. NHL:				NS
NHL	110 (95.7%)	93.2%	97.1%	
HD	5 (4.3%)	6.8%	2.9%	
Cell type:				NS
PBSC	110 (95.7%)	93.2%	97.1%	
Umbilical cord blood	5 (4.3%)	6.8%	2.9%	
Chronic GVHD overall score:				NS
Mild	56 (48.7%)	54.6%	46.4%	
Moderate	44 (38.3%)	38.6%	36.2%	
Severe	15 (13.0%)	6.8%	17.4%	
Time to cGHVD post HCT (Days, median)	173 [IQR 122;295]			
Time to CT scan post HCT (Days, median)	77 [IQR 33;202]			
Prior acute GVHD:				
No	19 (16.5%)	11.4%	18.8%	NS
Yes	96 (83.5%)			
KPS ≥ 90 at baseline:	86 (75.4%)			
KPS ≥ 90 at cGVHD onset:	65 (57.0%)	56.8%	56.5%	NS
HCT‐CI ≥ 3 at baseline:	38 (33.6%)	15(34.1%)	23(33.3%)	NS
HCT‐CI ≥ 3 at cGVHD onset:	61 (54.0%)	27(61.4%)	33(47.8%)	NS
Platelet at cGVHD onset: (X10^3^/μL)	145 ± 79	145 ± 78	144 ± 81	NS
Bilirubin at cGVHD onset: (mg/dL)	0.63 ± 0.88	0.57 ± 0.61	0.66 ± 1.07	NS
Weight on admission: (kg)	79.2 ± 16.4	68.6 ± 11.8	86.0 ± 15.4	< 0.001
Weight at cGVHD onset: (kg)	73.0 ± 14.2	63.0 ± 9.5	79.4 ± 12.9	< 0.001
BMI on admission: (kg/m^2^)	27.0 ± 4.6	25.8 ± 4.6	27.8 ± 4.5	0.022
BMI at cGVHD onset	24.9 ± 3.8	23.6 ± 3.6	25.7 ± 3.8	0.005
BMI ≥ 30 on admission	29 (25.2%)	15.9%	33.3%	0.032
BMI ≥ 30 at cGVHD onset	12 (10.5%)	4.5%	14.5%	0.12
Body composition at cGVHD onset				
SMI (cm^2^/m^2^)	40.9 ± 8.9	34.3 ± 6.0	45.1 ± 7.8	< 0.001
Fat index (cm^2^/m^2^)	100.0 ± 46	95.3 ± 42	103.1 ± 49	NS
% visceral fat	41 ± 14	33.4 ± 11.7	45.9 ± 13.3	< 0.001
High fat index (≥ 35% body fat)	28 (24.8%)	18.2%	29%	0.141
High fat index, optimal cutoff	70 (61.9%)	47.7%	71.0%	0.011
Sarcopenia (Kubrak)^a^				< 0.001
No sarcopenia		6.8%	49.3%	
Class I sarcopenia		50%	31.9%	
Class II sarcopenia		43.2%	18.8%	
Muscle radiodensity (HU)	35.7 ± 7.5	34.7 ± 7.8	36.3 ± 7.3	NS
Mean change in body composition from HCT to cGVHD (*n* = 69)				
SMI change from HCT‐cGVHD	−4.18 ± 5.19	−3.07 ± 3.95	−5.04 ± 5.88	0.118
Fat Index change from HCT‐cGVHD	−7.44 ± 20.8	−5.16 ± 18.3	−9.19 ± 21.4	0.410

^a^ Published cutoff values from Kubrak et al. [32].

### 3.2 Body Composition and Arthrometric Measures

Since weight and body composition are sex‐specific, data are presented for male and females separately (Table 1). Weight and body mass index were recorded at onset of cGVHD, and abdominal CT imaging at a median of 77 days 77 [IQR 33;202] after HCT was evaluated to assess body composition. While none of the demographic and clinical variables mentioned above differed by sex, there were distinctive differences between men and women in weight and body composition. Men were significantly heavier in overall body weight, had higher BMI and were more likely to be obese (defined as BMI ≥ 30 kg/m^2^). CT imaging revealed that the men also had higher fat index values, as well as a higher proportion of visceral adipose tissue than women. The distinctive features among females in this sample was significantly lower weight and BMI than males with a high rate of sarcopenia (93.2% overall including 50% with moderate sarcopenia and 43.2% with severe sarcopenia) compared with males (50.7% overall including 31.9% with moderate sarcopenia and 18.8% with severe sarcopenia (*p* < 0.001, Chi^2^ test).

**FIGURE 2:**
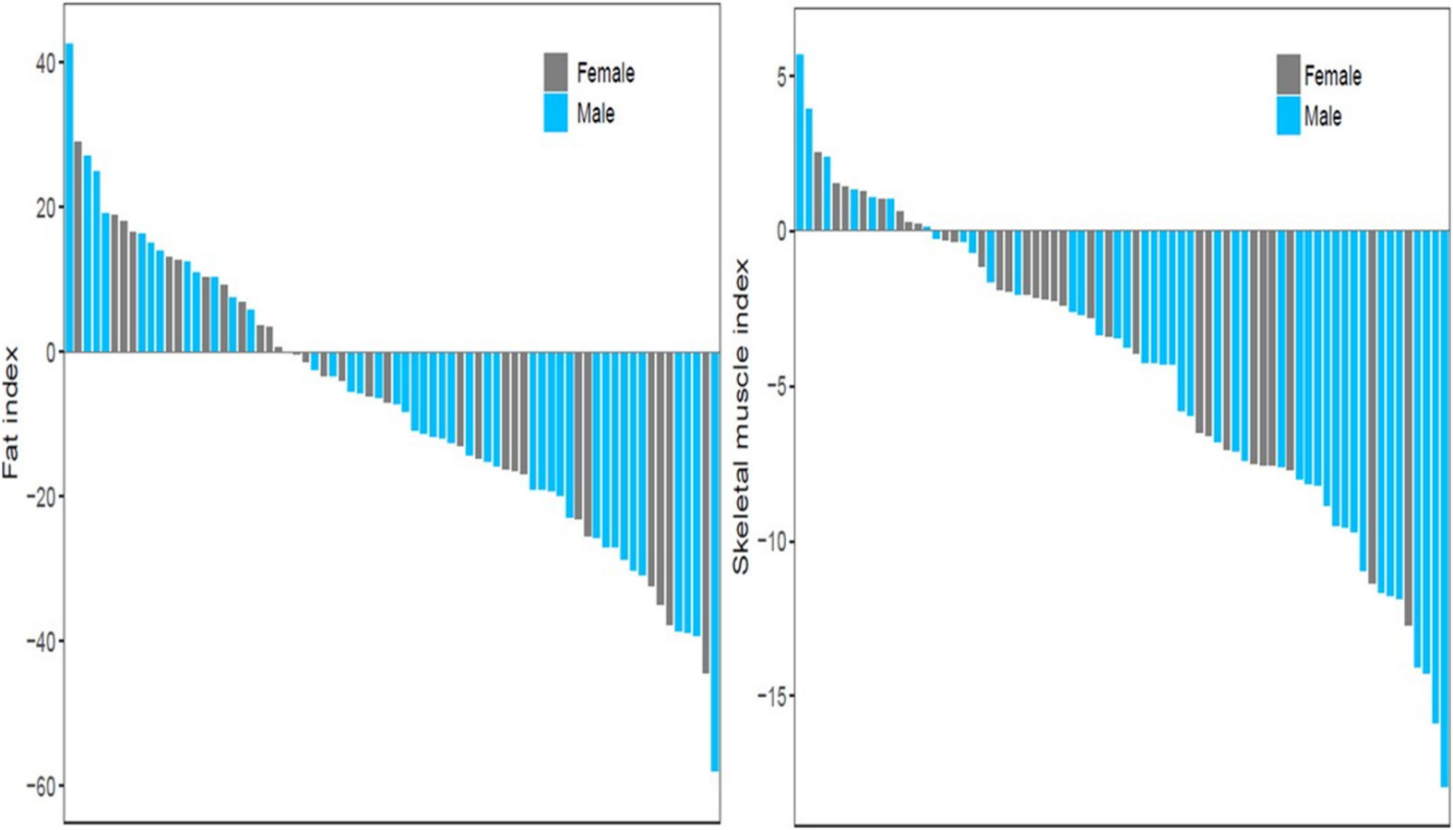
Waterfall plot illustrating individual patient‐level change in fat index (FI) and skeletal muscle index (SMI) from baseline pre‐HCT CT scan to post‐HCT CT scan pre‐dating cGVHD onset.

The body composition at onset of cGVHD was in part defined by change in SMI and FI that occurred since the transplant and this was analysable in a subset of *n* = 69 patients with available CT images pre‐ and post‐transplant (Table 1). The mean overall change of SMI in females was −8.2% and in males −9.9% (NS), an intense rate of loss over an interval of ~100 days. These losses were of great magnitude compared to treatment‐related muscle loss in other Oncology settings [32]. This change showed a wide range of intra‐individual variability. While most patients lost both SMI and FI during this interval, about 22.7% of patients overall maintained or gained SMI and 35.7% maintained or gained FI (Figure 2).

### 3.3 Survival Analysis

**FIGURE 3:**
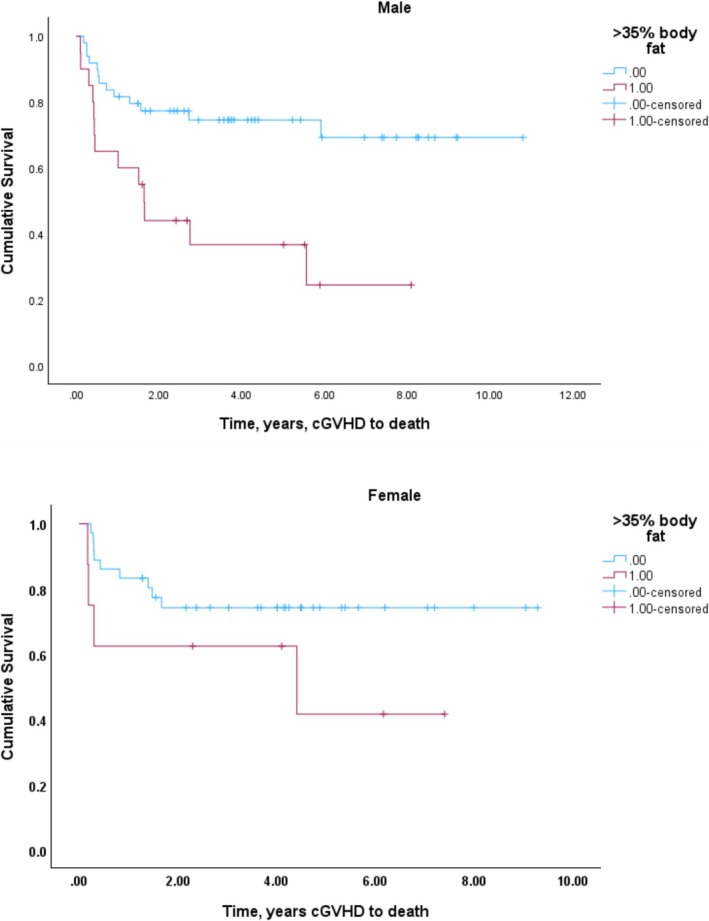
Univariable association between body fat ≥ 35% and overall survival from onset of chronic Graft Versus Host Disease (cGVHD). Upper panel: Males with body fat ≥ 35% (blue), Log Rank *p* < 0.001. Lower panel: Females with body fat ≥ 35% (blue), Log Rank *p* < 0.125.

The median overall follow up was 12 years, during which time there were 39 deaths. In the primary analysis examining features at cGVHD onset for association with overall survival (Table 2a), univariate analysis demonstrated that age (*p* = 0.014), platelet count (*p* = 0.003), and KPS ≥ 90 (*p* = 0.023) associated with survival. Levels of bilirubin, HCT‐CI, overlap vs. classic cGVHD status, cGVHD Grade were not associated with OS and were not included in the multivariable analysis. Among the body composition variables (Table 2), neither body mass index, nor skeletal muscle index/sarcopenia associated with survival at the univariable level. A radiological measure of high adiposity, total FI above the optimal cutoff (male: 66 cm^2^/m^2^; female: 88 cm^2^/m^2^) was associated with increased hazard for mortality. However, while this association was seen for the *n* = 70 patients defined as having high absolute fat index, the mortality associated with a subset of *n* = 28 patients whose high fat index value resulted in an overall body fat ≥ 35%. While present in both male and female, this effect was more prominent in males (Figure 3). Men with body fat ≥ 35% showed early death with 8/19 (42%) mortality within 1 year of developing cGVHD; 3/8 (37%) of females with body fat ≥35% also died within a year of developing cGVHD.

**TABLE 2:** Association of cGVHD and L3 body composition variables with overall survival outcome. Univariate (A) and multivariate (B) analysis of overall survival.

A:			
Variable	Level	HR (95% CI)	*p*
Age		1.045 (1.010, 1.081)	0.008
Platelet at cGVHD onset		0.993 (0.989, 0.998)	0.003
Bilirubin at cGVHD onset		1.145 (0.806, 1.626)	0.490
KPS at cGVHD onset	< 90	1.0 (Reference)	—
	≥ 90	0.480 (0.255, 0.905)	0.023
BMI at cGVHD onset		1.064 (0.977, 1.157)	0.153
HCT‐CI at cGVHD onset	< 3	1.0 (Reference)	—
	≥ 3	1.34 (0.72, 2.50)	0.360
Overlap vs. classic cGVHD	Classic and de novo	1.0 (Reference)	—
	Late acute and overlap	1.15 (0.62, 2.14)	0.655
Chronic GVHD overall score at onset	Mild	1.0 (Reference)	—
	Moderate	0.623 (0.298, 1.302)	0.208
	Severe	1.801 (0.791, 4.097)	0.161
SMI continuous		0.989 (0.954; 1.026)	0.559
Sarcopenic obese (Prado)		1.216 (0.375; 3.11)	0.684
Fat index, cm^2^/m^2^	Continuous	1.003 (0.996; 1.009)	0.390
Muscle radiodensity, HU	Continuous	0.946 (0.907, 0.986)	0.011
High fat index (≥ 35% body fat)	No	1.0 (Reference)	—
	Yes	2.994 (1.561, 5.552)	<0.001
High fat index, cm^2^/m^2^	No, categorical by optimal cutoff	1.0 (Reference)	—
	Yes	2.789 (1.282, 6.072)	0.010

B1: Multivariate analysis of OS			
Variable	Level	HR (95% CI)	*p*
Age	Continuous	1.058 (1.016, 1.102)	0.006
Platelet at cGVHD onset	Continuous	0.992 (0.988, 0.997)	< 0.002
KPS at cGVHD onset	Continuous	0.953 (0.926, 0.977)	0.001
High fat index (≥35% body fat)	No	1.0 (Reference)	—
	Yes	2.094 (1.060, 4.136)	0.033

B2 Additional multivariable models: Indices of fatness			
Variable^a^	Level	HR (95% CI)	*p*
BMI, kg/m^2^	Continuous	1.064 (0.977, 1.157)	0.153
Sarcopenic obese (Prado)	Categorical	0.951 (0.357, 2.528)	0.919
Fat index, cm^2^/m^2^	Continuous	1.001 (0.994, 1.008)	0.735
High fat index, cm^2^/m^2^	No, categorical by optimal cutoff	1.0 (Reference)	—
	Yes	2.024 (0.902, 4.534)	0.087
Skeletal muscle, HU	Continuous	1.010 (0.957,1.066)	0.716

^a^ Adjusted for age, platelet at cGVHD onset and KPS at cGVHD onset.

Owing to the limited number of events, multivariable analysis was constrained to 4 variables in total.

In the final multivariable model (Table 2Bi), age, platelet count, KPS and body fat ≥ 35% remained significantly associated with overall survival at least a *p* < 0.05 level, while other indices of adiposity including BMI, sarcopenic obesity, high fat index defined by optimal stratification, and muscle radiodensity were not significant.

### 3.4 Secondary Analysis of Chest CT‐Based Body Composition

While study of radiographic body composition at L3 represents the gold standard in the field, chest CT scans are much more commonly performed for several reasons (disease re‐staging, investigation of pulmonary symptoms or infections) in a broader group of patients with HCT. Thus, to enhance feasibility and dissemination of this approach for future studies, we also examined body composition variables using the T4 slice of thoracic CT scans. However, we note the T4 analysis does not completely recapitulate that performed at L3, as fat index at T4 is missing visceral fat. As presented in Table 3, univariate analysis demonstrated similar effects for each studied body composition variable as the L3 studies. However, in multivariable analysis, FI demonstrated similar effect but not at *p* value < 0.05.

**TABLE 3:** Association of cGVHD and T4 body composition variables with overall survival outcome. Univariate (A) and Multivariate (B) Analysis of Overall Survival.

A: Univariate analysis			
Variable	Level	HR (95% CI)	*p*
Age		1.05 (1.01, 1.08)	0.005
Platelet at cGVHD onset		0.99 (0.99, 1.00)	0.002
Bilirubin at cGVHD onset		1.04 (0.75, 1.43)	0.822
KPS at onset of cGVHD	< 90	1.0 (Reference)	—
	≥ 90	0.45 (0.25, 0.82)	0.009
Weight at second scan (kg)		1.00 (0.98, 1.02)	0.758
HCT‐CI at cGVHD onset	< 3	1.0 (Reference)	—
	≥ 3	1.44 (0.79, 2.62)	0.238
Overlap vs. classic cGVHD	Classic and de novo	1.0 (Reference)	—
	Late acute and overlap	1.16 (0.65, 2.08)	0.612
Chronic GVHD overall score	Mild	1.0 (Reference)	—
	Moderate	0.65 (0.33, 1.28)	0.215
	Severe	1.51 (0.70, 3.26)	0.292
SMI	≤ Optimal cutoff	1.0 (Reference)	—
	> Optimal cutoff	0.86 (0.48, 1.55)	0.616
FI	≤ Optimal cutoff	1.0 (Reference)	—
	> Optimal cutoff	2.10 (1.12, 3.94)	0.021
SMI HU	≤ Optimal cutoff	1.0 (Reference)	—
	> Optimal cutoff	0.43 (0.22, 0.81)	0.009

B: Multivariate analysis			
Variable	Level	HR (95% CI)	*p*
Age		1.07 (1.03, 1.11)	< 0.001
Platelet at cGVHD onset		0.99 (0.99, 1.00)	< 0.001
KPS at onset of cGVHD	< 90	1.0 (Reference)	—
	≥ 90	0.52 (0.28, 0.97)	0.038
FI	≤ Optimal cutoff	1.0 (Reference)	—
	> Optimal cutoff	1.86 (0.97, 3.59)	0.064

## 4 Discussion

New tools are needed to assess mortality risk among patients with cancer treated with intensive therapies. To address this need in arguably one of the most vulnerable cancer populations [33], we analysed the association of radiographic body composition with overall survival among patients with cancer with cGVHD after HCT. Our prior work has demonstrated the high prevalence of sarcopenia in HCT recipients determined by both radiographic and functional measurements prior to even beginning HCT‐related treatment. This analysis presented herein fulfils the major goal of providing evaluation of patients after HCT when they have recovered from their acute peri‐transplant toxicities and begin their long‐term care for chronic risks including cGVHD. Our study provides insight into the association of CT‐based radiographic body composition measures with survival after cGVHD diagnosis (which has not been examined to date), and ultimately once validated could be considered in further refinement of currently NIH Consensus cGVHD severity assessment as well as a risk stratification tool for novel interventional studies.

Conventional methodology to evaluate body habitus by weight and BMI has been utilized in the HCT setting [34]; however, these methods are unable to identify the composite contributions of lean and fat tissue in a patient population (cancer) known to have highly variable distribution of both muscle and fat [35]. The primary analysis based on L3 radiographic data provided important results, where increased adiposity was independently associated with inferior survival outcome, adjusting for other established prognostic variables in this setting. While the role of obesity on transplant related outcomes has been described and indexed to determine prognosis, the role of fat mass or skeletal muscle independently has only recently been explored [36, 37]. As we explored the contribution of fat distribution itself, we have been able to examine the role of fat independently [38, 39]. Fat index was sexually dimorphic with male patients having greater adiposity than females, and in this sample both men and women reached a fat mass ≥ 35% of overall body weight and it was this subset, which showed an independent association with early mortality. Our data suggest that low skeletal muscle mass alone does not predict reduced survival in cGVHD as previously described in other cancers. Longitudinal reports note the protective role of low BMI in patients with extensive cGVHD suggestive of the role of fat in patients with this chronic morbid condition [40]. We note that validation of these findings is needed, and should be based in a large, independent, likely multi‐centre study population. Beyond this initial analysis, further study in larger populations may provide adequate power to more completely discern the impact of high fat index on mortality.

To enhance feasibility and dissemination, secondary analysis of chest CT‐based body composition measures was performed. The results largely recapitulate those from the abdominal scans, thus opening opportunities for future study of larger and more representative HCT populations. L3 muscle structures include the internal, external, and transverse abdominus, psoas, and quadratus lumborum whereas for muscles for respiration (T4) are recruited primarily involved the internal intercostals, intercostalis intimi, and subcostals. We previously reported moderate correlation for skeletal muscle parameters between the regions, but higher correlation for total adipose tissue prior to HCT between the two. Changes in individual tissue compartments and unequal distribution of tissue loss may be important when comparing L3 and T4 regions, with adipose tissue at the abdominal level includes visceral adipose tissue in comparison to the chest where this is absent [33]. Again, additional study in larger patient populations may help elucidate this effect further. We note that CT‐based assessment of fat and muscle mass is not affected by pulmonary parenchymal abnormalities; hence, CT scans obtained for work up of infectious issues frequently encountered post‐HCT could facilitate future large studies of chest CT‐based body composition assessment.

We intentionally focused on radiographic body composition measures available near the time of chronic GVHD onset, given our primary interest in this population. We anticipated, however, that dynamic changes in body composition would occur in the trajectory from pre‐HCT to later time points post‐HCT as well. Examining our assembled study population, we found that only a subset (*n* = 69) had paired pre‐HCT and post‐HCT scans within our stated window, thus we considered any further investigation in this to be fully exploratory. The dominant trend was that of decrease in FI and SMI over time. We propose that future investigation is needed to more fully characterize the effect of change in FI and SMI over time, with attention to much larger study populations. However, overall these data demonstrate intense FI and SMI loss over time. Muscle loss in the peri‐HCT period is equal to values seen in other challenging treatment plans such as neoadjuvant chemo‐radiation in oesophageal cancer or metastatic pancreatic cancer treated with FOLFIRINOX [32]. This muscle loss is equivalent to about 25 years of aging, compressed into about 3 months, so that a 50‐year‐old person at HCT, has the muscle mass of a 75 year old following transplant. HCT offers a chance at long‐term survival; however, for many patients, this period begins with profound sarcopenia. Post‐HCT, the challenge is rehabilitating the muscle atrophy/pathology considering the expectation of long‐term survival. Sarcopenia is associated with reduced physical functioning, and if this persists after HCT, it is an important detriment to long‐term health and mobility.

We note the following limitations: First, there are limitations to our available study population. This retrospective analysis was necessarily limited to those patients that had CT scans performed in routine practice near the onset of cGVHD, thus limiting it to HCT recipients with lymphoma. With this in mind, we cannot generalize these findings to much more common HCT indications, such as acute leukaemia and myelodysplastic syndrome. Single‐centre and registry data support that the majority of HCT procedures are done for these indications, and they are not represented in our current study. As well, utilization of allogeneic HCT for lymphoma has been and will likely continue to be relatively low, given more frequently used therapeutic procedures such as autologous transplant and more recently CAR‐T. Allogeneic HCT has been used for salvage therapy after failure of CAR‐T, thus opening up other potential areas of study, yet volumes remain relatively low at the present time. Our population was further limited to those with cGVHD and also those with available CT imaging studies for analysis. We acknowledge this leads to convenience sampling rather than population‐based estimates for the studied body composition measures. Second, we note that CT scans were selected given their availability and proximity to cGVHD onset, however these were not prospectively performed exactly at time of cGVHD onset. Given the overall trajectory of change in body composition we observed from the pre‐HCT to post‐HCT, ongoing changes may well be occurring from day 100 post‐HCT to roughly 5–6 months post‐HCT, the expected median onset time of cGVHD. Thus, our use of these scans is an approximation based on available studies only. This limitation again speaks to the need for well‐designed prospective studies with measures obtained at time of chronic GVHD onset. Third, we note the relatively limited sample size in this initial study. These initial findings require validation, and larger studies will likely be able also to refine conclusions sarcopenia (SMI, HU), as well as utilization of chest CT studies instead. Larger studies would also be needed to accomplish a goal of refining prognostic sub‐groups within currently defined NIH cGVHD severity categories using body composition data, whereas our findings are considered exploratory. We note that prior larger scale studies in the field have established the expected distribution of overall chronic GVHD severity, and that overall moderate chronic GVHD (a relatively large group comprised of mixed organ involvement and severity patterns) would represent an excellent target for leveraging body composition measures to further risk stratify such patients. Finally, we acknowledge that the cohort examined is historic, and that HCT itself, cGVHD incidence, cGVHD therapy, and supportive care continue to evolve into the modern era. Our study sample had transplant in the 2005–2016 range, which spans a large period of changes, advances in HCT technology, and improved outcomes overall. We note prior work, which has demonstrated improved survival over time per transplant era. Even from 2016 onward to present day, major shifts are occurring, most notably improved GVHD prevention strategies, which have resulted in decreased incidence of chronic GVHD. Future studies will need to include more contemporaneous cohorts to fully address these concerns.

## Conflicts of Interest

The authors declare no conflicts of interest.
